# Event-Related Potentials

**Published:** 1995

**Authors:** Shirley Y. Hill

**Affiliations:** Shirley Y. Hill, Ph.D., is professor of psychiatry, psychology, and human genetics at the Western Psychiatric Institute and Clinic, University of Pittsburgh Medical Center, Pittsburgh, Pennsylvania

**Keywords:** brain, evoked potential, high-risk youth, AOD dependence, biological markers, hereditary factors

**Figure:**
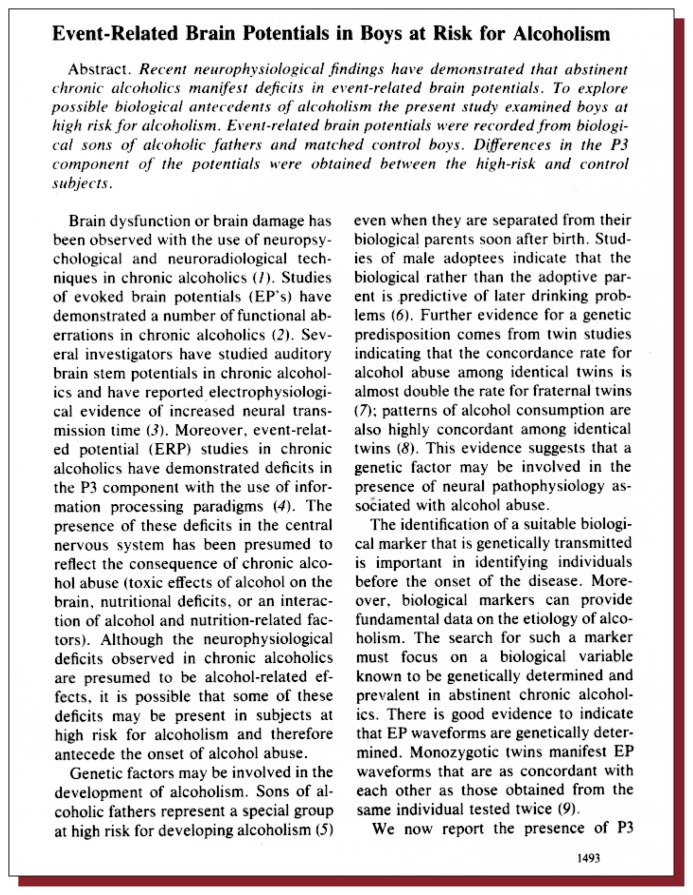
Begleiter, H.; Porjesz, B.; Bihari, B.; and Kissin, B. Event-related brain potentials in boys at risk for alcoholism. *Science* 225(4669):1493–1496, 1984.

Alongstanding and extensive body of research exists discussing the cognitive impairment seen in chronic alcoholics, starting with the classic work of Victor and colleagues (1971), who described changes in the brain and nervous system seen on autopsies of alcohol-dependent subjects. The changes observed in the brain were correlated with both memory and attentional deficits. The idea that cognitive changes, as reflected in scores on neuropsychological tests given to alcoholics, were associated with the causes rather than the consequences of alcohol use was conceptually appealing (Goodwin and Hill 1975).

P300 has received much attention as a possible neurophysiological risk marker for the development of alcoholism. The event related potential (ERP) is a series of waves that appear on a scan of the brain’s electrophysiological activity following an auditory or visual stimulus. P300, one of the waves that compose the ERP, peaks approximately 3/10ths of a second after an informative event occurs. Alterations in the height of the P300 wave (i.e., its amplitude) in some studies may be a cause or a consequence of drinking (Porjesz et al. 1980). Solving the puzzle of the possible relationship between P300 and drinking is especially difficult because a multitude of factors exist that affect the emergent ERP waveform.

## P300 as a Risk Marker for Alcohol Problems

For more than a decade, researchers have examined the possibility that the P300 component of the ERP may have etiological significance for alcoholism (Pfefferbaum et al. 1979,^1^ Begleiter et al. 1984; Hill et al. 1987). However, P300’s importance to alcohol research is only now being fully appreciated, as more studies from different laboratories support its usefulness as a predictor of later alcohol problems.

This landmark article by Begleiter and colleagues (1984) captured the scientific community’s interest because the smaller P300 amplitude seen in this study was in 12-year-old boys at risk for developing alcoholism due to their family histories. The researchers recorded P300 in 25 sons of alcoholics and compared their patterns with those of 25 control subjects, finding differences in the P300 wave recordings which suggested that the P300 decrement in sons of alcoholics was an indicator of inherited risk for alcoholism and not the result of alcohol use. Later research has shown that uncovering the source of the P300 decrease in amplitude in subjects at risk for developing alcohol problems because of their family background depends on critical age, gender, and sensory (visual or auditory) systems.

## P300 Amplitude Correlates With Development

Because the P300 component is one measure of a person’s cognitive capacity, both the amplitude of the P300 peak and the time between peaks (its latency) have been studied in subjects differing in some aspect of cognition or behavior or in some degree of maturation. For example, P300 amplitude and latency do change as children develop (Howard and Polich 1985) and according to the varying rates at which children’s auditory and visual processes mature (Courchesne et al. 1983). It has been shown that the neural generators of P300 (i.e., the parts of the brain that power the P300) may differ for each sensory system, visual or auditory (Johnson 1989*a*, *b*). Other key parameters such as age, drinking history, and gender also must be considered when P300 amplitude is used as a risk marker (Steinhauer and Hill 1993; Hill and Steinhauer 1993*a*).

## Heritability of P300 Amplitude

Much evidence exists suggesting that brain neuroelectrical activity, including ERP’s, is heritable (O’Connor et al. 1994). A greater similarity in ERP waves is observed between immediate family members than between unrelated individuals (Steinhauer et al. 1987). Data on alcoholic families tested for inheritance patterns for the P300 component suggest the presence of a major gene controlling the similarity in P300 amplitude (Aston and Hill 1990).

## P300 Amplitude in Alcoholics May Vary by Gender

Although not all studies have found such differences (Pfefferbaum et al. 1979; Steinhauer and Hill 1987; Hill et al. in press*a*), characteristic low-amplitude P300 peaks have been reported for middle-age abstinent male alcoholics when compared with control subjects (Pfefferbaum et al. 1991; Porjesz et al. 1987*a*,*b*). However, adult female alcoholics show profound reductions in P300 amplitude when compared with age-matched nonalcoholic controls (Hill and Steinhauer 1993*b*). Future research will show whether P300 differences among high- and low-risk boys and girls disappear or persist into adulthood.

## P300 in High-Risk Children

Despite inconsistencies in the research on P300 as a risk marker for alcoholism in adulthood, several laboratories now have documented differences in P300 characteristics between high- and low-risk children (Begleiter et al. 1984; Hill et al. 1990; Steinhauer and Hill 1993; Hill and Steinhauer 1993*a*; Berman et al. 1993). In their seminal article, Begleiter and colleagues pioneered the use of P300 as a potential marker for the development of alcoholism. More recent studies have determined that not all high-risk (FHP; i.e., family history positive) children have the marker; but then, of course, not all high-risk children develop alcohol problems. Approximately one-third of high-risk boys and one-fifth of high-risk girls have been found to display P300 amplitude reduction (Steinhauer and Hill 1993).

## P300 Pattern’s Relationship to Clinical Outcome

Two recent followup studies found increased rates of alcohol and other drug abuse and dependence among adolescents from FHP backgrounds who showed reduction of P300 when they first were evaluated as children (Berman et al. 1993; Hill et al. in press*b*). Berman and colleagues (1993) found that those subjects who had the lowest P300 values when they were evaluated at age 12 had significantly increased rates of alcohol and other drug abuse when reevaluated at age 16. Hill and colleagues (in press*b*) completed a followup of a group of high- and low-risk subjects initially tested for ERP characteristics in 1985 at age 10, who were retested and evaluated clinically after approximately 8 years. The ERP testing was repeated with the same paradigm, and continued reduction of the P300 amplitude was apparent. Significantly more of the high-risk subjects met criteria for alcohol and other drug dependence than did the low-risk subjects. Moreover, the subjects, now 18 years old, who developed alcohol problems had significantly lower P300 than did those without alcohol problems.

In summary, that ERP waveforms appear heritable suggests that P300 amplitude could be transmitted from parent to offspring. Because FHP offspring may inherit both the tendency to develop alcoholism and a tendency to have a lower amplitude P300, the P300 may be one index of genetic vulnerability to alcohol dependence. Although further research is needed to validate this risk marker for alcoholism, the evidence to date points to its usefulness as a childhood predictor of alcohol and other drug use and dependence. The landmark study by Begleiter and colleagues was key in starting a whole series of investigations looking at this possibility.

## Footnotes

1 In their 1979 research, Pfefferbaum and colleagues did not observe changes in P300 amplitude among alcoholics but did see changes in P300 latency (i.e., the time between peaks).
